# Diagnostic Yield of *APOL1 p.N264K* Variant Screening in Daily Practice

**DOI:** 10.1016/j.ekir.2024.04.008

**Published:** 2024-04-04

**Authors:** Céline Narjoz, Vinh-Hoang-Lan Julie Tran, Marion Rabant, Alexandre Karras, Nicolas Pallet

**Affiliations:** 1 Department of Clinical Chemistry, Assistance Publique Hôpitaux de Paris, Georges Pompidou European Hospital, Paris, France; 2 Department of Pathology, Assistance Publique Hôpitaux de Paris, Necker Hospital, Paris, France; 3 University Paris Cité, Paris, France; 4 Department of Nephrology, Assistance Publique Hôpitaux de Paris, Georges Pompidou European Hospital, Paris, France

**Keywords:** *APOL1*, chronic kidney disease, *p.N264K*

## Introduction

Large American cohorts of individuals of African ancestry have demonstrated a strong protective effect of the *p.N264K* variant of *APOL1* on the risk of chronic kidney disease, focal segmental glomerulosclerosis, and end-stage kidney disease.^1^^,^^2^ This variant is only effective in the presence of the high-risk, African, G2 haplotype, and is never observed in patients carrying the high-risk G1/G1 genotype. The protective effect of this variant seems to be related to the reversal of the ability of the mutant APOL1 protein to form ionic conductance pores.^2^^,^^3^

*APOL1* high-risk variants testing has a role in identifying people at higher risk of developing kidney disease for more aggressive management of risk factors, counseling of potential kidney donors, and selection of candidates for therapies targeting APOL1.^4^^,^^5^ Determination of *p.N264K* status may be an important component of *APOL1* diagnostic testing, and therefore support routine implementation in high-risk status (G1/G2 and G2/G2) patients. Indeed, the reclassification of high-risk patients to low-risk could have a direct impact on subsequent clinical decision making, particularly in the etiologic investigation and management of kidney disease, the selection of living donors, and the identification of candidates for therapies targeting high-risk APOL1.

However, the profile of patients screened for *APOL1* high-risk variants in daily clinical practice is likely very different from the populations in which the protective role of *p.N264K* has been established; and before implementing a policy of screening for this variant in routine clinical practice, a number of factors need to be considered. The frequency of the *p.N264K* variant in the Million Veteran Program, an observational cohort study in the Department of Veterans Affairs health care system, is estimated to be 6% in the G1/G2 and G2/G2 high-risk genotypes, but only 0.4% in the whole cohort^2^; and a precise estimate of the expected prevalence of the variant in a highly specific population of patients with kidney disease and candidate for *APOL1* screening is important to consider when introducing an additional genetic target into clinical routine, particularly in terms of cost effectiveness. Moreover, for routine implementation, it is also important to know what the mutational profile is in populations other than those initially described, such as African-Europeans. Finally, it is important to understand the pathologic phenotype of patients carrying the protective variant, within a population of unselected individuals referred for exploration of kidney disease of various causes.

## Results

Since 2016, our department has been screening for high-risk *APOL1* variants in all patients who originate from sub-Saharan Africa and the French West Indies and who are being investigated for kidney disease. As of December 2023, 629 patients were genotyped for *APOL1* variants G1 (p.S342G; p.I384M) and G2 (p.NYK388K, a 6 base pair deletion removing amino acids N388 and Y389) based on their African origin (detailed methods can be found in the Supplementary Material). The strategy in our department is to perform *APOL1* genotyping in all patients referred for investigation of kidney disease, regardless of cause, symptomatology, or severity, as long as they are of African origin. Kidney biopsies are performed to a much lesser extent, and indications are broad but not systematic in the presence of kidney disease.

**Figure 1:**
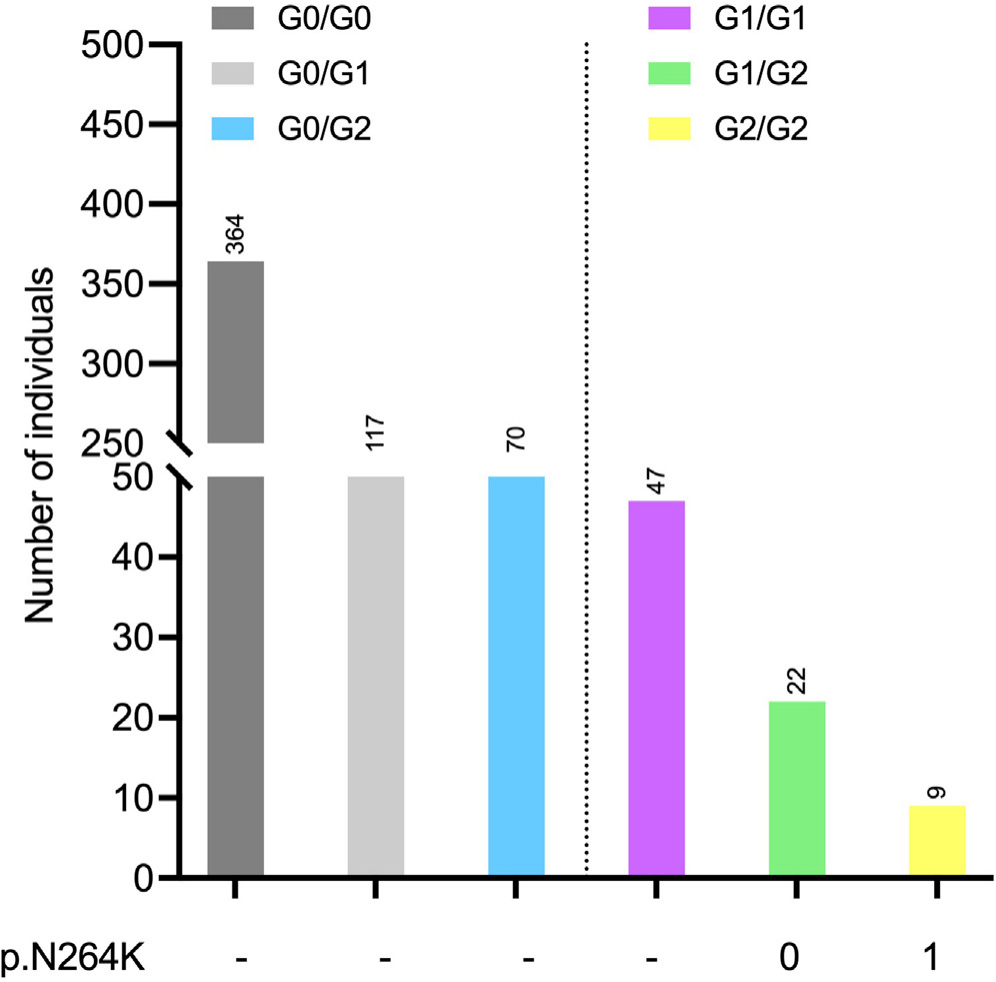
Distribution of APOL1 genotypes in the 629 patients of sub-Saharan and West Indian origin who underwent kidney biopsy. P-N264K variant was retrospectively screened in the G1/G2 and G2/G2 carriers.

Of these, 78 (12.4%) were classified at high risk: 47 G1/G1 (7.4%), 22 G1/G2 (3.8%), and 9 G2/G2 (0.9%) (Figure 1). We retrospectively genotyped the *APOL1 p.N264K* in the 31 high-risk G1/G2 and G2/G2 patients. We identified only 1 patient with a single copy of the *p.N264K* allele representing 3.2% of high-risk G1/G2 and G2/G2 patients, and 1.2% of high-risk patients when including G1/G1. This single patient was G2/G2 homozygous. This result compares with data from the Million Veteran Program cohort (121,492 participants of African descent, 15% with chronic kidney disease), in which 579 carriers of a *p.N264K* variant were observed out of 9695 G1/G2 and G2/G2 carriers (i.e., 5.9%) and 15,752 high-risk genotypes including G1/G1 (3.6%).^2^ The prevalence of high-risk alleles in the genotyped population is relatively low. We cannot exclude the possibility that “African-European” populations differ slightly from “African-American” populations in terms of *APOL1* genetics. Indeed, the frequency of the high-risk allele in sub-Saharan Africa varies widely between populations and countries.^6^ For example, in Senegal and Cameroon, where a large proportion of the African patients followed-up with in France come from, the frequency of these risk alleles is about 5%, compared to Nigeria, where it can be as high as 45%. Finally, the indications for APOL1 genotyping in our patients do not reflect the severity of kidney disease, because all patients of African origin are genotyped. This highlights the fact that high-risk *APOL1* mutations are not necessarily the cause of kidney disease.

We next determined whether the *p.N264K* variant might be associated with a specific phenotype in the 31 G1/G2 and G2/G2 patients group. Kidney biopsy was not performed in 8 patients. One case was uninterpretable (absence of glomeruli). The description of the 22 G1/G2 and G2/G2 cases with a conclusive kidney biopsy is shown in Table 1. The pathologic diagnoses in the high-risk group were as follows: focal segmental glomerulosclerosis (*n* = 7), lupus nephritis (*n* = 4), HIV-associated nephropathy (*n* = 3), COVID-associated nephropathy (*n* = 2), IgA nephropathy (*n* = 2), tubulointerstitial nephropathy–associated sarcoidosis (without glomerular lesion) (*n* = 2), membranous nephropathy (*n* = 1) and cryoglobulinemia-associated glomerulonephritis (*n* = 1). Critically, the only strictly normal biopsy was found on the carrier of the *p.N264K* variant (G2/G2). This 39-year-old patient was evaluated for proteinuria of the order of 1 g/g and microhematuria without kidney dysfunction. Immunofluorescence was normal, as were COL4A3, 4, and 5 chains staining. A random association cannot be excluded; however, its probability can be considered low, given the rarity of the *p.N264K* variant in the general population.

**Table 1:** Characteristics of the 22 G1-G2 and G2-G2 patients who underwent kidney biopsy

Age	Sex	Initial presentation	Underlying disease	APOL1 status	sCr (micomol/L)	Proteinuria (g/g)	Hematuria (Y/N)	Pathologic diagnosis	FSGG lesion (Y/N)
45	F	Proteinuria	HIV	G1-G2, N264K-	350	1.6	Y	HIVAN	Y
17	F	NS	NA	G1-G2, N264K-	105	6.2	N	FSGS (primitive)	Y
36	M	Proteinuria	NA	G2-G2, N264K-	110	4	N	FSGS (primitive)	Y
37	M	Proteinuria	Lupus	G1-G2, N264K-	67	2	Y	LN III+V	N
47	F	Proteinuria	NA	G1-G2, N264K-	77	3.4	Y	FSGS (secondary)	Y
50	M	CKD	Hypertension	G1-G2, N264K-	184	2.5	Y	FSGS (secondary)	Y
25	M	Proteinuria	HIV	G1-G2, N264K-	107	1.2	N	HIVAN	Y
48	M	NS	HIV	G1-G2, N264K-	225	4.4	Y	HIVAN	Y
26	M	RPGN	Lupus	G1-G2, N264K-	280	3.1	Y	LN class III	Y
35	F	AKI on CKD	Sarcoidosis	G1-G2, N264K-	170	0.6	N	Tubulointerstitial nephritis	N
59	F	CKD	Sarcoidosis	G2-G2, N264K-	604	0.4	N	Tubulointerstial nephritis	N
35	M	Proteinuria	NA	G2-G2, N264K+	89	1.3	Y	Normal	N
74	M	CKD	Diabetes	G1-G2, N264K-	114	1 .1	N	DN	Y
35	F	NS-AKI	Hypertension	G1-G2, N264K-	229	10.3	N	FSGS (secondary)	Y
67	M	NS-AKI	COVID	G1-G2, N264K-	310	8.2	Y	COVAN	Y
28	M	CKD3A	Obesity	G1-G2, N264K-	251	2.5	Y	FSGS (secondary)	Y
69	M	CKD3A	Hypertension	G2-G2, N264K-	210	2.1	Y	IgA nephropathy	Y
75	F	AKI	Diabetes, COVID	G1-G2 N264K-	192	2.7	N	COVAN	Y
60	F	Proteinuria	Hypertension	G1-G2, N264K-	82	1.1	N	MN	Y
45	F	CKD	Heart trasplantation (tacrolimus/everolimus)	G1-G2, N264K-	126	3.4	N	FSGS (secondary)	Y
43	F	RPGN	NA	G1-G2	225	4	Y	Cryoblobulinemia-MPGN	N
41	F	RPGN	Lupus	G2-G2	81	1.5	Y	LN III	Y

AKI, acute kidney injury; CKD, chronic kidney disease; COVAN, COVID-associated nephropathy; DN, diabetic nephropathy; F, female; FSGS, focal segmental glomerulosclerosis; HIVAN, HIV-associated nephropathy; LN, lupus nephritis: membranoproliferative glomerulonephritis; M, male; MN, membranous nephropathy; N, no; NA, nonavailable; NS, nephrotic syndrome; RPGN, rapidly progressive glomerulonephritis; TIN, tubulointerstial nephritis; Y, yes.

## Discussion

We describe the results of screening for the *p.N264K* variant in a cohort of patients with kidney disease and referred to a tertiary care center. The size of our series is small compared to the large cohorts that have described the protective role of this variant, but it provides practical information for daily practice in a nephrology department that performs more than 300 kidney biopsies per year and requests nearly 80 *APOL1* genotypes per year for patients of sub-Saharan African or West Indian origin (who represent about 30% of our patient population).

It is debatable whether it is worth genotyping more than 600 individuals to find 1 case (in other words, 1 case in 8 years); however, if limited to G1/G2 and G2/G2 patients, this represents a nonnegligible proportion (3.2%) with clinical or therapeutic consequences. For example, at the individual level, this screening is of considerable interest in certain situations, such as the evaluation of kidney donors. In some countries, such as France, *APOL1* screening is recommended and living kidney donation is contraindicated for high-risk polymorphism carriers.^7^ Therefore, the identification of the protective variant could maintain access to donation for a significant number of potential candidates. The cost-effectiveness ratio is the corollary of this first observation, and will depend essentially on test reimbursement policies. Finally, we observed that the only patient with a normal biopsy is the one carrying the protective variant. Obviously, a larger cohort needs to be analyzed and a larger genotype-phenotype correlation needs to be performed to reach a statistically valid conclusion.

## Disclosure

All the authors declared no competing interests.

## Supplementary Material

Supplementary File (PDF) **Supplementary Methods. Supplementary References**.

## Footnotes

Supplementary File (PDF)

**Supplementary Methods.**

**Supplementary References.**
