# Supplementary material for: Diagnostic Yield of APOL1 p.N264K Variant Screening in Daily Practice
Source: Kidney Int Rep. 2024 Apr 4;9(6):1916–8. doi: 10.1016/j.ekir.2024.04.008 (PMC11184237; doi:10.1016/j.ekir.2024.04.008)
Supplement: Supplementary File (PDF) — Supplementary Methods. Supplementary References. [file mmc1.pdf]

Supplementary methods

Patients

HEGP Kidney Disease cohort: Between January 2016 and December 2023, 2484 consecutive individuals were referred to the Nephrology Department at the Georges Pompidou European Hospital (Paris, France) for a kidney biopsy, including 1908 for a native kidney biopsy. Indications for biopsy are at the discretion of the nephrologist but respond to the need to make a diagnosis (estimated GFR < 60ml/min and/or proteinuria > 0.5 g/l and/or non urologic hematuria and/or acute kidney injury and/or acute kidney disease), to monitor the activity of a disease or the response to a treatment. Kidney biopsies were only for patient care. Detailed information regarding the clinical, medical, demographic, biological, and histological status of the patients was collected using an information-based data warehouse. The patients were informed of the study and did not object to the use of their clinical and biological data collected within the framework of the care exclusively. Data management complies with French reference methodologies. All patient were informed of the participation in the study and provided a signed consent form for the genetic analysis. Protocol approval was obtained from the ethic committee CERAPHP (Comité d'éthique de la recherche AP-HP Centre), registration number 00011928.

APOL1 genotyping

APOL1 was performed in the Clinical Chemistry Department of HEGP in Paris, France in all patients from sub-saharan africa and the West indies referred for a kidney biopsy. Peripheral whole blood was collected from each patient at the time of inclusion during a regular monitoring of the disease. DNA was extracted from leucocytes using the kit Maxwell_16 LEV Blood DNA (Promega, France), according to the manufacturer instructions. Genotyping was performed on a QuantStudio 6 and 7 using Taqman assays (Applied Biosystems, Thermo Fisher Scientific). G1 polymorphism was defined by the presence of the two mutations rs73885319 and rs60910145, or mutations rs73885319 and the G2 one was defined by the mutation rs71785313. The *APOL1* allele with the rs73885319 mutation without the rs60910145 mutation was considered a high-risk allele [S1].

Supplementary reference

S1. Kopp JB, Nelson GW, Sampath K, et al. APOL1 Genetic Variants in Focal Segmental Glomerulosclerosis and HIV-Associated Nephropathy. J Am Soc Nephrol. 2011;22(11):2129-2137. doi:10.1681/ASN.2011040388
